# Predicting Long-term Outcomes in Deceased Donor Kidney Transplant Recipients Using Three Short-term Graft Characteristics

**DOI:** 10.34067/KID.0000000000000154

**Published:** 2023-05-22

**Authors:** Shaifali Sandal, Marcelo Cantarovich, Heloise Cardinal, Agnihotram V. Ramankumar, Lynne Senecal, Suzon Collette, Chee Long Saw, Steven Paraskevas, Jean Tchervenkov

**Affiliations:** 1 Division of Nephrology, Department of Medicine, McGill University Health Centre, Montreal, Quebec, Canada; 2 Research Institute of the McGill University Health Centre, Montreal, Quebec, Canada; 3 Multiorgan Transplant Program, Departments of Medicine and Surgery, McGill University Health Centre, Montreal, Quebec, Canada; 4 Division of Experimental Medicine, McGill University Health Centre, Montreal, Quebec, Canada; 5 Department of Medicine, University of Montreal, Montreal, Quebec, Canada; 6 Department of Medicine, Hôpital Maisonneuve-Rosemont, Montreal, Quebec, Canada; 7 Division of Hematology, Department of Medicine, McGill University Health Centre, Montreal, Quebec, Canada; 8 Department of Surgery, McGill University Health Centre, Montreal, Quebec, Canada

**Keywords:** delayed graft function, graft outcomes, patient outcomes, acute kidney injury, 90-day outcomes

## Abstract

**Key Points:**

- Delayed graft function is not an ideal measure of graft function, yet is used to assess risk in kidney transplantation.
- We propose a model that combines it with two other measures of 90-day graft function to identify recipients at incremental risk of inferior long-term outcomes.

**Background:**

Delayed graft function (DGF) in kidney transplant recipients is used to determine graft prognosis, make organ utilization decisions, and as an important end point in clinical trials. However, DGF is not an ideal measure of graft function. We aimed to develop and validate a model that provides incremental risk assessment for inferior patient and graft outcomes.

**Methods:**

We included adult kidney-only deceased donor transplant recipients from 1996 to 2016. In addition to DGF, two short-term measures were used to assess risk: renal function recovery <100% (attaining half the donor's eGFR) and recipient's 90-day eGFR <30. Recipients were at no, low, moderate, or high risk if they met zero, one, two, or all criteria, respectively. Cox proportional hazard models were used to assess the independent relationship between exposure and death-censored graft failure (DCGF) and mortality.

**Results:**

Of the 792 eligible recipients, 24.5% experienced DGF, 40.5% had renal function recovery <100%, and 6.9% had eGFR <30. Over a median follow-up of 7.3 years, the rate of DCGF was 18.7% and mortality was 25.1%. When compared with recipients at no risk, those at low, moderate, and high risk were noted to have an increase in risk of DCGF (adjusted hazard ratio [aHR], 1.53; 95% confidence interval [CI], 1.03 to 2.27; aHR, 2.84; 95% CI, 1.68 to 4.79; aHR, 15.46; 95% CI, 8.04 to 29.71) and mortality (aHR, 1.16; 95% CI, 0.84 to 1.58; aHR, 1.85; 95% CI, 1.13 to 3.07; aHR, 2.66; 95% CI, 1.19 to 5.97). When using a hierarchical approach, each additional exposure predicted the risk of DCGF better than DGF alone and 100 random bootstrap replications supported the internal validity of the risk model. In an external validation cohort deemed to be at lower risk of DCGF, similar nonsignificant trends were noted.

**Conclusion:**

We propose a risk model that provides an incremental assessment of recipients at higher risk of adverse long-term outcomes than DGF alone. This can help advance the field of risk assessment in transplantation and inform therapeutic decision making in patients at the highest spectrum of inferior outcomes.

## Graphical abstract

**Figure:**
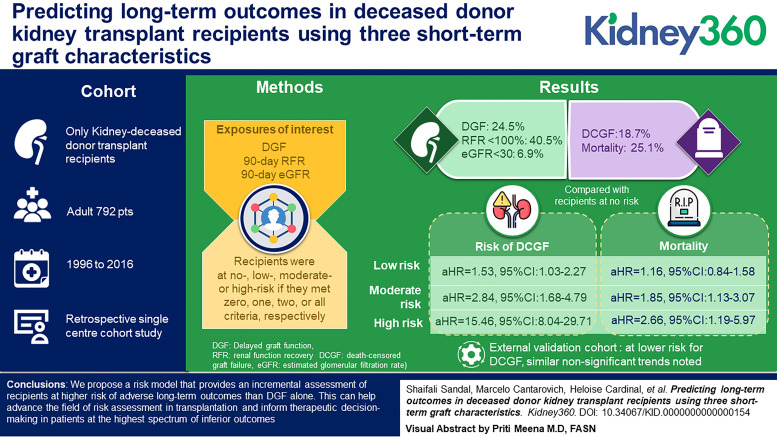

## Introduction

In kidney transplantation, risk assessment to guide therapeutic decision making and inform clinical practice is a critical area of research. Indeed, several prediction models for graft failure have been developed and validated.^1^ These models, however, tend to focus on donor and recipient characteristics before transplantation as predictors of outcomes after transplantation.^1,2^ Even the Kidney Allocation System only accounts for pretransplant characteristics of the donor and kidney transplant recipient (KTR).^3^ Limited work has been performed in developing and validating risk models that incorporate early measures of graft function as a predictor of adverse long-term graft outcomes.

Graft ischemia and then reperfusion is a form of acute kidney injury.^4,5^ Lack of recovery or partial recovery after acute kidney injury is widely recognized to be associated with inferior outcomes in the general nephrology literature.^6–15^ Currently, one measure of graft recovery that is universally captured is delayed graft function (DGF).^16^ DGF is associated with reduced graft survival in some, but not all types of donors.^17–23^ Although definitions of DGF are not standardized,^16,24^ the most widely accepted definition is the need for dialysis in the first 7 days. This assessment is subjective, clinician and center-dependent, and does not necessarily capture graft dysfunction.^16,25^ In addition, changes in the first week after transplantation may be because of perioperative issues and may not offer a reliable assessment of graft function. The 90-day landmark, an important quality metric in the general nephrology and surgical literature, may instead be a more logical time frame to assess graft function.^26–28^

Given these limitations of DGF measurements, others have explored changes in eGFR over the first few months and reported them to be associated with outcomes after transplantation.^2,23,29–32^ We have also developed a short-term outcome called renal function recovery (RFR) and reported that the lack of attaining at least half the donor's eGFR after kidney transplantation is associated with a 45% higher risk of graft failure.^33^ However, these binary measures do not provide an incremental assessment of risk. Identification of patients at the highest spectrum of inferior outcomes may guide retransplantation-related decisions and CKD and comorbidity management.

Thus, we aimed to develop and validate a risk prediction model in KTRs that uses three short-term measures of graft function. We postulate that this model that incorporates functional assessment of a graft over the first 90 days can better stratify a recipient's risk of graft failure and mortality than DGF alone.

## Methods

### Study Cohorts

This was a retrospective cohort study of adult kidney-only deceased donor transplant recipients at the McGill University Health Centre from January 1, 1996, to March 30, 2016. Those with graft failure or death within 90 days and those who were lost to follow-up were excluded. Patients who received more than one kidney transplantation within the study period were censored at the time of the second transplantation. Finally, we excluded donors with terminal serum creatinine cutoff ≥133 umol/L. This cutoff was chosen on the basis of one of the criteria used to define expanded criteria donors (ECDs). In addition, these patients likely had acute kidney injury; hence, the true donor's eGFR and RFR could not be reliably calculated.

For external validation, we chose a relatively lower-risk sample of KTRs from two neighboring transplant centers. This cohort comprised a dataset created for another study at the University of Montreal. Briefly, this cohort included 809 KTRs from the Centre Hospitalier de l'Université de Montréal and the Hôpital Maisonneuve-Rosemont between June 1, 2008, and December 31, 2016. Deidentified data were obtained after appropriate ethics approval. This study was approved by the Research Ethics Board at the McGill University Health Centre.

### Risk Model

The three exposures of interest were DGF, 90-day RFR, and 90-day eGFR <30 ml/min per 1.73 m^2^. The risk model is presented in Table 1. DGF was defined as the need for dialysis within the first week after transplantation. RFR was calculated as $\frac{Observed\;eGFR}{Predicted\;eGFR}\times 100$. Observed eGFR was calculated using the average of the best three eGFR values within the first 3 months after transplantation. The predicted recipient eGFR was half the donor's terminal eGFR, plus 15, if the recipient was a preemptive KTR. The terminal or last donor serum creatinine before organ procurement was used to estimate the donor's GFR. The Chronic Kidney Disease Epidemiology Collaboration prediction equation was used to determine eGFR and is here on expressed as ml/min per 1.73 m^2^.^34^

**Table 1:** Risk prediction model: Based on a literature review, three short-term measures of graft function that are independently associated with graft outcomes were used to develop a model to stratify kidney transplant recipients at an incremental increase in risk of adverse long-term outcomes

Level of Risk	Characteristic (RFR<100% or DGF or 90-d eGFR<30)
No risk	None
Low risk	1 of the above
Medium risk	2 of the above
High risk	All of the above

RFR, renal function recovery; DGF, delayed graft function.

### Outcome Measurements

Outcomes of interest were death-censored graft failure (DCGF), all-cause graft failure (ACGF), and mortality. KTRs were censored at graft loss (return to dialysis or retransplantation), death, or end of the study period (December 31, 2018), whichever came first. We also wanted to test the validity of our model to predict CKD stage ≥4 (eGFR<30 or graft loss) at the 1-, 5-, and 10-year marks.

### Statistical Analyses

The distributions of the recipient, donor, and transplant characteristics at transplantation were evaluated by DGF, RFR, and eGFR using the *t*-test or Kruskal-Wallis test for continuous variables and the chi square or Fisher exact test for categorical variables. Univariable and multivariable Cox proportional hazard models were used to assess the independent relationship between the exposure and the outcomes of interest. We used Cox proportional hazard models to analyze the association between our risk prediction model and outcomes. We used a graded increase in the risk score to assess the relationship for each outcome.

For internal and external validation, the outcome of interest was DCGF. Because DGF is the most prominent outcome captured in the transplant literature, we wanted to attempt a hierarchical modeling approach of the added predictive value of 90-day RFR and 90-day eGFR. To ensure the robustness of our analysis, we conducted a sensitivity analysis in a cohort of KTRs who received an ECD and also those who received a transplant in a more recent era (2006–2016). We used the bootstrapping technique to ensure that the model calibration derived from our sample is not overtly optimistic and to obtain a more realistic (bias-corrected) calibration.^35^ We used 100 random bootstrap replications to get bias-corrected calibration in our prediction model. Finally, we performed an external validation of our risk model for the outcome of DCGF.

Statistical analyses were performed using Stata/IC 14.2(StataCorp, College Station, TX, www.stata.com) and R version 3.4.2 (R Foundation for Statistical Computing, Vienna, Austria, www.R-projects.org). Missing covariate data were handled by multiple imputations using Stata's ice command and R's mice package. A two-tailed *P*-value of <0.05 was considered statistically significant.

## Results

**Figure 1:**
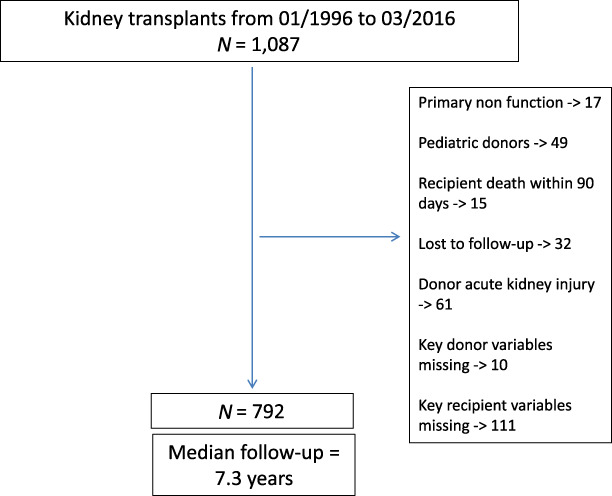
Study flow diagram.

During the study period, 792 KTRs were eligible for analysis, of which 24.5% experienced DGF (Figure 1). The mean RFR was 116, and 6.9% had a 90-day eGFR of <30. Baseline characteristics of the total cohort and stratified by DGF are presented in Table 2 and stratified by the RFR and eGFR are presented in Supplemental Tables 1 and 2, respectively. Of note, 42% of our cohort received grafts from ECDs.

**Table 2:** Baseline characteristics of the total cohort and stratified by delayed graft function

Characteristic	Total Cohort (*N*=792)	No DGF (*n*=598)	DGF (*n*=194)
**Recipient characteristics**			
Age, mean (SD)	55.4 (13.1)	55.7 (12.9)	54.5 (13.5)
Female sex, %	34.1	34.8	32
BMI in kg/m^2^, mean (SD)	27.0 (5.2)	26.8 (5.1)	27.8 (5.2)
White race, %	68.4	69.4	65.5
Type of dialysis, %			
*Hemodialysis*	81.3	77.1	94.3
*Peritoneal dialysis*	6.8	7.7	4.1
*Preemptive*	10.7	13.9	1.0
*Missing*	1.1	1.3	0.5
Months on dialysis, mean (SD)	17.9 (13.9)	15.7 (9.6)	34.2 (28.5)
Cause of end-stage kidney disease, %			
*Diabetes*	25.3	24.2	28.5
*Polycystic kidney disease*	13.2	13.5	13.1
*Glomerulonephritis*	31.3	32.0	29.0
*Hypertension*	11.0	11.1	10.9
*Other*	19.2	19.6	18.1
Cold ischemia time, h (SD)	16.8 (6.6)	16.8 (6.5)	16.9 (6.9)
Warm ischemia time, h (SD)	0.8 (0.3)	0.8 (0.3)	0.8 (0.3)
Machine perfusion, %	48.0	48	47.9
**Donor characteristics**			
Age, mean (SD)	52.7 (14.6)	51.7 (14.9)	55.8 (13.2)
Female sex, %	47	48.3	42.8
BMI, mean (SD)	27.2 (9.0)	27.1 (9.8)	27.4 (5.9)
White race, %	83.0	84.3	78.9
ECD, %	42.0	39.3	50.5
Donation after cardiac death, %	7.6	5.2	15
Terminal eGFR, mean (SD)	99.1 (21.3)	99.7 (21.3)	97.4 (21.1)
Induction immunosuppression, %			
*Antithymocyte globulin*	55.7	55.2	57.2
*Alemtuzumab*	33.8	32.8	37.1
*Interleukin-2 receptor inhibitor*	8.8	9.2	7.7
Maintenance immunosuppression, %			
*Tacrolimus*	84.1	84.8	82
*Cyclosporine*	8.2	7.3	10.8
*Sirolimus*	1.3	1.2	1.6
*Mycophenolate*	84.7	84.8	85.5
*Azathioprine*	5.1	4.7	6.2
*Prednisone*	60.4	61.4	57.2
RFR, mean (SD)	116.3 (47.9)	119.1 (48)	107.6 (46.8)
90-d eGFR <30, %	6.9	3.9	16.5

DGF, delayed graft function; BMI, body mass index; ECD, expanded criteria donor; RFR, renal function recovery.

### Baseline Demographics Stratified by Exposures of Interest

Those who experienced DGF were more likely to be on hemodialysis, spend more time on dialysis before transplantation, and receive grafts from ECDs and donors with circulatory determination of death. In addition, when compared with KTRs who did not have DGF, those with DGF were noted to have a lower RFR (119 versus 107.6) and were more likely to have an eGFR of <30 (3.9% versus 16.5%) (Table 2).

KTRs with lower RFR were more likely to have received machine perfusion and grafts from ECDs, donors with circulatory determination of death, and donors with lower terminal eGFR. In those with RFR >100%, 75%–100%, and ≤75%, DGF was noted in 21.4%, 21.8%, and 38% of KTRs, respectively. In addition, 0%, 1.7%, and 36.6% had eGFR <30, respectively, in those categories (Supplemental Table 1).

Finally, when compared with KTRs who achieved an eGFR of ≥30, those with eGFR <30 were more likely to be older, male, and White patients. In addition, they were more likely to have received grafts from ECDs and donors with lower terminal eGFR. The mean RFR was lower in the cohort with eGFR <30 (121.2 versus 50.9), and more KTRs experienced DGF (22.0% versus 58.2%) (Supplemental Table 2).

### Risk of Graft Failure

Over a median follow-up of 7.3 years, 18.7% experienced DCGF and 37.1% experienced ACGF. DGF, lower RFR, and lower 90-day eGFR were all associated with a higher risk of DCGF and ACGF. The risk model, however, provided an incremental risk assessment. When compared with no risk, KTRs at low risk, moderate risk, and high risk had an adjusted hazard ratio (aHR) of 1.53 (95% confidence interval [CI], 1.03 to 2.27), 2.84 (95% CI, 1.68 to 4.79), and 15.46 (95% CI, 8.04 to 29.71), respectively. Similarly, for the outcome of ACGF, an incremental risk was noted: low risk: aHR, 1.28 (95% CI, 1.00 to 1.67); moderate risk: aHR, 1.88 (95% CI, 1.27 to 2.81); and high risk: aHR, 6.17 (95% CI, 3.68 to 10.35) (Table 3).

**Table 3:** Hazard ratio for death-censored graft failure, all-cause graft failure, and mortality by various exposures of interest

Exposure	Univariable Cox Proportional Hazard Model	Multivariable Cox Proportional Hazard Model^a^
DCGF		
DGF		
*No DGF*	1.00	1.00
*DGF*	2.44 (1.75–3.41)^b^	1.94 (1.36–2.77)^b^
RFR		
*>100%*	1.00	1.00
*75%–100%*	1.63 (1.10–2.42)^b^	1.51 (1.00–2.28)
*<75%*	2.62 (1.77–3.87)^b^	2.27 (1.51–3.41)^b^
90-d eGFR		
*≥*30	1.00	1.00
*<30*	7.73 (4.99–11.9)^b^	6.28 (3.91–10.11)^b^
Risk score		
*None*	1.00	1.00
*Low*	1.73 (1.18–2.53)^b^	1.53 (1.03–2.27)^b^
*Medium*	3.50 (2.14–5.74)^b^	2.84 (1.68–4.79)^b^
*High*	20.0 (11.04–36.20)^b^	15.46(8.04–29.71)^b^
ACGF		
DGF		
*No DGF*	1.00	1.00
*DGF*	1.86 (1.45–2.38)^b^	1.60 (1.23–2.08)^b^
RFR		
*>100%*	1.00	1.00
*75%–100%*	1.39 (1.05–1.84)^b^	1.32 (1.00–1.76)
*<75%*	1.70 (1.26–2.30)^b^	1.48 (1.08–2.01)^b^
90-d eGFR		
*≥30*	1.00	1.00
*<30*	4.38 (3.01–6.37)^b^	3.84 (2.55–5.67)^b^
Risk score		
*None*	1.00	1.00
*Low*	1.41 (1.10–1.82)^b^	1.28 (1.00–1.67)
*Medium*	2.15 (1.47–3.12)^b^	1.88 (1.27–2.81)^b^
*High*	9.43 (5.80–15.34)^b^	6.17 (3.68–10.35)^b^
Mortality		
DGF		
*No DGF*	1.00	1.00
*DGF*	1.62 (1.19–2.21)^b^	1.50 (1.09–2.10)^b^
RFR		
*>100%*	1.00	1.00
*75%–100%*	1.10 (0.75–1.52)	1.08 (0.76–1.56)
*<75%*	1.37 (0.94–2.00)	1.19 (0.80–1.76)
90-d eGFR		
*≥30*	1.00	1.00
*<30*	2.74 (1.58–4.76)^b^	2.66 (1.50–4.72)^b^
Risk score		
*None*	1.00	1.00
*Low*	1.23 (0.91–1.66)	1.16 (0.84–1.58)
*Medium*	1.81 (1.14–2.88)^b^	1.85 (1.13–3.07)^b^
*High*	4.13 (1.88–9.10)^b^	2.66 (1.19–5.97)^b^

DCGF, death-censored graft failure; DGF, delayed graft function; RFR, renal function recovery; ACGF, all-cause graft failure.

a Adjusted for recipient age, sex, race, body mass index, type of dialysis, history of hypertension and diabetes, machine perfusion, donor age, and donor sex.

b Significant values.

### Risk of Mortality

Over the follow-up period, 25.1% mortality was noted, and DGF and lower 90-day eGFR were associated with higher risk. Even here, the risk model provided an incremental risk assessment: low risk: aHR, 1.16 (95% CI, 0.84 to 1.58); moderate risk: aHR, 1.85 (95% CI, 1.13 to 3.07); and high risk: aHR, 2.66 (95% CI, 1.19 to 5.97) (Table 3).

### Graft Function

At 1 and 5 years, all exposures of interest were associated with a risk of CKD stage ≥4. DGF was associated with an aHR of 2.95 (95% CI, 1.84 to 4.71), RFR <75 was associated with an aHR of 5.76 (95% CI, 3.17 to 10.5), and eGFR <30 was associated with an aHR of 49.9 (95% CI, 65.2 to 381.9) for the outcome of CKD ≥4 at 5 years, in a multivariable analysis. The risk model also performed well. At 5 years when compared with no risk, KTRs at low, moderate, and high risk had an aHR of 1.78 (95% CI, 0.98 to 3.28), 5.17 (95% CI, 2.45 to 10.92), and 40.13 (95% CI, 5.07 to 317.8) for CKD ≥4 in a multivariable analysis, respectively. At 10 years, no graft with 90-day eGFR <30 survived, and compared with DGF and RFR, the risk model performed better at predicting CKD ≥4 for those deemed to be at low and moderate risk (Supplemental Table 3).

### Sensitivity Analysis and Internal Validation

When analyzing only those KTRs who received ECDs and those who received transplants between 2006 and 2016, similar trends were noted. When compared with no risk, KTRs at low, moderate, and high risk had an aHR of 1.36 (95% CI, 0.75 to 2.47), 2.75 (95% CI, 1.38 to 5.45), and 11.11 (95% CI, 5.11 to 24.15) for DCGF in multivariable analysis, respectively. When using a hierarchical approach, each additional exposure predicted the risk of DCGF better than DGF alone. When using 100 random bootstrap replications, similar results were noted to be supporting the internal validity of our risk model (Table 4).

**Table 4:** Internal and external validation: Hazard ratio for death-censored graft failure in a cohort of only those kidney transplant recipients who received expanded criteria donors, using a hierarchical modeling approach and 100 random bootstrap replications in the internal cohort and in an external cohort from two different transplant centers

Sub-Cohorts	Univariable Cox Proportional Hazard Model	Multivariable Cox Proportional Hazard Model^a^^,^^b^
ECD recipients only		
None	1.00	1.00
Low	1.57 (0.86–2.76)	1.36 (0.75–2.47)
Medium	3.26 (1.62–6.53)^c^	2.90 (1.37–6.14)^c^
High	13.62 (6.52–28.4)^c^	13.94 (5.77–31.27)^c^
KTRs (2006–2016 only)		
None	1.00	1.00
Low	1.69	(0.88–3.24)
Medium	2.32 (1.14–4.74)^c^	1.41 (0.39–4.05)
High	19.85 (8.17–48.23)^c^	9.64 (2.52–36.82)^c^
Hierarchical modeling approach		
No DGF	1.00	1.00
Only DGF	1.42 (0.77–2.63)	1.32 (0.67–2.63)
DGF+RFR<100%	1.98 (0.95–4.12)	1.87(0.83–4.20)
DGF+RFR<100%+eGFR<30	10.43 (5.38–20.2)^c^	9.35 (4.39–19.91)
Risk score and bootstrap with 100 random replications		
None	1.00	1.00
Low	1.73 (1.20–2.49)^c^	1.53 (1.05–2.24)^c^
Medium	3.50 (2.22–5.53)^c^	2.84 (1.62–4.95)^c^
High	20.0 (11.3–35.4)^c^	15.46 (7.83–30.55)^c^
External validation		
None	1.00	1.00
Low	2.28 (0.82–6.34)	2.50 (0.89–7.14)
Medium	2.51 (0.42–11.00)	2.88 (0.33–10.71)
High	NA	NA

ECD, expanded criteria donor; KTRs, kidney transplant recipients; DGF, delayed graft function; RFR, renal function recovery; NA, not applicable.

a Adjusted for recipient age, sex, race, body mass index, type of dialysis, history of hypertension and diabetes, and machine perfusion, donor age, and donor sex.

b In the external validation cohort, given the low number of events, we only adjusted for recipient age, sex, race, history of diabetes, machine perfusion, donor age, and donor sex.

c Significant values.

### External Validation

Of the 809 transplantations performed during the study period, 672 KTRs were eligible for analysis. Reasons for exclusion were lack of availability of terminal donor creatinine (*n*=46), terminal donor creatinine ≥133 umol/L (*n*=28), pediatric donor (*n*=29), primary nonfunction or death in <3 months (*n*=14), and other key variables missing (*n*=20). In addition, the 3-month creatinine measurements were not available; hence, 6-month measurements were used to assess RFR and eGFR. In this cohort, the average recipient age was lower (50.9) than the main cohort, and the proportion of non-White patients (19.6%), patients with diabetes (14.1%), and those who received grafts from ECDs (33.4%) were also lower. In addition, there were more preemptive transplantations (13.8%) (Supplemental Table 4). Thus, the DGF rate was much lower (11.9%), and over a median follow-up of 5.1 years, the DCGF rate was only 4.3%. Of note, DGF was not associated with DCGF (aHR, 2.31; 95% CI, 0.66 to 8.06). Although no recipient fit the definition of high risk, in the low-and moderate-risk categories, the risk model demonstrated a nonsignificant but increased risk of DCGF.

## Discussion

In this study, we developed and validated a risk prediction model using three 90-day measures of graft function and stratified KTRs at low, moderate, and high risk of adverse long-term patient and graft outcomes. When compared with KTRs who were deemed to be at no risk, those deemed to be at moderate and high risk were 2.8 and 15.5 times more likely to have DCGF, 1.9 and 6.2 times more likely to have ACGF, and 1.9 and 2.7 times more likely to die, respectively. The model was validated in a cohort of KTRs who received ECDs and used other internal validation approaches. In addition, using an external validation cohort, similar nonsignificant trends were noted. Overall, our risk prediction model that used three simple measures of graft function was a better prognostic tool than DGF alone and can help advance the field of risk assessment in transplantation.

In our cohort and in this literature, DGF was associated with a 1.4–1.9 times higher risk of graft failure.^36–38^ Our risk prediction model was able to identify KTRs at incremental and much higher risk of DCGF. This is likely because we incorporated two markers of graft function, *i.e.*, 90-day RFR and 90-day eGFR. These two markers are a better assessment of recovery after ischemic reperfusion injury of an explanted graft, irrespective of whether the KTR was diagnosed with DGF. Incomplete recovery or lack of recovery after acute kidney injury is almost universally acknowledged to be associated with adverse patient and renal outcomes in the general nephrology, medicine, and surgical literature.^7–14^ Our group and others have also shown that KTRs who cannot attain an eGFR of at least 30 have inferior graft outcomes, irrespective of whether they experienced DGF.^30,39^ Thus, we propose this model be considered in identifying KTRs at the highest risk of adverse graft and patient outcomes.

Although our model performed well in a high-risk cohort of KTRs who received ECDs, it did not reach significance in the external cohort. This external cohort was likely at lower risk of DCGF because the incidence of DGF was much lower and baseline characteristics that can put a graft at higher risk of failure were lower than our cohort as well. This finding can also be explained by the shorter follow-up and lower prevalence of DCGF, which may have limited the power to detect a statistically significant difference. In addition, no KTR met the high-risk category to test for the validity of the model. Regardless, similar trends were noted in this cohort.

Our model also identified KTRs at higher risk of mortality. Similar to our cohort, many studies have shown that DGF is associated with higher mortality.^36,38–40^ In an analysis of deceased-donor kidney transplantations from 1998 to 2004, DGF was associated with a 1.8 times higher risk of death with a functioning graft.^40^ In another analysis of KTRs from the Scientific Registry of Transplant Recipients database between 1997 and 2010, DGF was associated with 1.6 times higher risk of mortality.^36^ We identified recipients who were at 2.7 times higher risk of death. This observation follows general medicine and surgical literature where acute kidney injury is often a predictor of adverse patient outcomes, including mortality.^6–15^ This is likely due to a complex interplay of genetic, molecular, and immune-based mechanisms, in addition to baseline comorbidities.^41^ We postulate similar mechanisms in KTRs with DGF, RFR <100%, and eGFR <30.

The biggest strength of our study is that we used three predefined exposures that others have shown to independently predict graft function and/or survival. However, we combined them to identify KTRs at incremental risks of adverse outcomes. Using a minimal and readily available set of variables, we can identify patients at the highest risk spectrum, which has therapeutic implications. In addition, we used the 90-day landmark approach, which is not just an important quality metric in the surgical literature^26^ but is used to define CKD.^28^ We acknowledge the following limitations: Although our external validation cohort was from a different center, our findings need to be validated in other external cohorts that include KTRs of varied risk profiles. Similar to others, the need for dialysis after transplantation at our center is subjective; however, our DGF incidence and its predictive power were identical to what has been reported in the literature. We were unable to account for the effect of early rejection after transplantation. The observational nature of this study makes it vulnerable to residual confounding, and the small sample size and number of events limit our ability to adjust for all relevant confounders in the multivariable analysis.

Despite this, our findings are very relevant to the field of kidney transplantation. Notwithstanding the limitations of DGF mentioned earlier, it continues to be used as an important short-term measure to determine graft prognosis, to make organ utilization decisions, and as an important primary end point in clinical trials.^42,43^ Limitations of DGF have been outlined by many.^16,24,25^ Many interventions, such as machine perfusion, that decrease the risk of DGF do not affect long-term outcomes.^44^ Although we do not recommend abolishing DGF altogether, we instead recommend that to address dependent predictors and use them to create a risk prediction model, the 90-day landmark be considered.^1,45^ We recommend adding two 90-day short-term measures of graft function that can better risk stratify KTRs and thus serve as a better prognostic tool than DGF alone. This can aid early interventions and therapeutic decision making to improve the outcome of KTRs with varied risk profiles and could potentially be explored as an outcome measure in clinical trials.

In conclusion, we developed, compared, and validated a risk model that integrated DGF with two 90-day measures of graft function and report this model to be a better prognostic tool for determining long-term patient and graft outcomes than DGF alone. We also note that this model can help identify KTRs at the highest spectrum of graft failure and mortality, which can help guide post-transplant management strategies. By combining conventional wisdom from the general surgical and nephrology literature, we hope to have created a predictive model that can meaningfully influence the field of transplantation.

## Supplementary Material

SUPPLEMENTARY MATERIAL

## Data Availability

All data are included in the manuscript and/or supporting information.

## Supplemental Material

Supplemental Table 1. Baseline characteristics stratified by 90-day renal function recovery.

Supplemental Table 2. Baseline characteristics stratified by 90-day eGFR.

Supplemental Table 3. Logistic odds ratio for CKD stage ≥4 by various exposures of interest.

Supplemental Table 4. Baseline characteristics of the external validation cohort.

## References

[1] Kabore R Haller MC Harambat J Heinze G Leffondre K. Risk prediction models for graft failure in kidney transplantation: a systematic review. Nephrol Dial Transplant. 2017;32(suppl _2):ii68–ii76. doi:10.1093/ndt/gfw405 28206633

[2] Kasiske BL Israni AK Snyder JJ Skeans MA Peng Y Weinhandl ED. A simple tool to predict outcomes after kidney transplant. Am J Kidney Dis. 2010;56(5):947–960. doi:10.1053/j.ajkd.2010.06.020 20801565

[3] Stewart DE Kucheryavaya AY Klassen DK Turgeon NA Formica RN Aeder MI. Changes in deceased donor kidney transplantation one year after KAS implementation. Am J Transplant. 2016;16(6):1834–1847. doi:10.1111/ajt.13770 26932731

[4] Mannon RB. Delayed graft function: the AKI of kidney transplantation. Nephron. 2018;140(2):94–98. doi:10.1159/000491558 30007955 PMC6165700

[5] Sandal S Bansal P Cantarovich M. The evidence and rationale for the perioperative use of loop diuretics during kidney transplantation: A comprehensive review. Transplant Rev (Orlando). 2018;32(2):92–101. doi:10.1016/j.trre.2017.11.002 29242033

[6] McKown AC Wang L Wanderer JP, . Predicting major adverse kidney events among critically ill adults using the electronic health record. J Med Syst. 2017;41(10):156. doi:10.1007/s10916-017-0806-4 28861688 PMC5821255

[7] Bagshaw SM Laupland KB Doig CJ, . Prognosis for long-term survival and renal recovery in critically ill patients with severe acute renal failure: a population-based study. Crit Care. 2005;9(6):R700–R709. doi:10.1186/cc3879 16280066 PMC1414056

[8] Hsu CY Chertow GM McCulloch CE Fan D Ordonez JD Go AS. Nonrecovery of kidney function and death after acute on chronic renal failure. Clin J Am Soc Nephrol. 2009;4(5):891–898. doi:10.2215/CJN.05571008 19406959 PMC2676192

[9] Pannu N James M Hemmelgarn B Klarenbach S. Association between AKI, recovery of renal function, and long-term outcomes after hospital discharge. Clin J Am Soc Nephrol. 2013;8(2):194–202. doi:10.2215/CJN.06480612 23124779 PMC3562863

[10] Helgadottir S Sigurdsson MI Palsson R Helgason D Sigurdsson GH Gudbjartsson T. Renal recovery and long-term survival following acute kidney injury after coronary artery surgery: a nationwide study. Acta Anaesthesiol Scand. 2016;60(9):1230–1240. doi:10.1111/aas.12758 27378715

[11] Ishani A Nelson D Clothier B, . The magnitude of acute serum creatinine increase after cardiac surgery and the risk of chronic kidney disease, progression of kidney disease, and death. Arch Intern Med. 2011;171(3):226–233. doi:10.1001/archinternmed.2010.514 21325112

[12] Coca SG King JT Jr. Rosenthal RA Perkal MF Parikh CR. The duration of postoperative acute kidney injury is an additional parameter predicting long-term survival in diabetic veterans. Kidney Int. 2010;78(9):926–933. doi:10.1038/ki.2010.259 20686452 PMC3082138

[13] Coca SG Singanamala S Parikh CR. Chronic kidney disease after acute kidney injury: a systematic review and meta-analysis. Kidney Int. 2012;81(5):442–448. doi:10.1038/ki.2011.379 22113526 PMC3788581

[14] Chawla LS Bellomo R Bihorac A, . Acute kidney disease and renal recovery: consensus report of the Acute Disease Quality Initiative (ADQI) 16 workgroup. Nat Rev Nephrol. 2017;13(4):241–257. doi:10.1038/nrneph.2017.2 28239173

[15] Silver SA Harel Z McArthur E, . Causes of death after a hospitalization with AKI. J Am Soc Nephrol. 2018;29(3):1001–1010. doi:10.1681/ASN.2017080882 29242248 PMC5827605

[16] Yarlagadda SG Coca SG Garg AX, . Marked variation in the definition and diagnosis of delayed graft function: a systematic review. Nephrol Dial Transplant. 2008;23(9):2995–3003. doi:10.1093/ndt/gfn158 18408075 PMC2727302

[17] Boom H Mallat MJ de Fijter JW Zwinderman AH Paul LC. Delayed graft function influences renal function, but not survival. Kidney Int. 2000;58(2):859–866. doi:10.1046/j.1523-1755.2000.00235.x 10916111

[18] Nagaraja P Roberts GW Stephens M, . Influence of delayed graft function and acute rejection on outcomes after kidney transplantation from donors after cardiac death. Transplantation. 2012;94(12):1218–1223. doi:10.1097/tp.0b013e3182708e30 23154212

[19] Singh RP Farney AC Rogers J, . Kidney transplantation from donation after cardiac death donors: lack of impact of delayed graft function on post-transplant outcomes. Clin Transplant. 2011;25(2):255–264. doi:10.1111/j.1399-0012.2010.01241.x 20331689

[20] Summers DM Johnson RJ Allen J, . Analysis of factors that affect outcome after transplantation of kidneys donated after cardiac death in the UK: a cohort study. Lancet. 2010;376(9749):1303–1311. doi:10.1016/s0140-6736(10)60827-6 20727576

[21] Lim WH McDonald SP Russ GR, . Association between delayed graft function and graft loss in donation after cardiac death kidney transplants—a paired kidney registry analysis. Transplantation. 2017;101(6):1139–1143. doi:10.1097/TP.0000000000001323 28538652

[22] Wadei HM Heckman MG Rawal B, . Comparison of kidney function between donation after cardiac death and donation after brain death kidney transplantation. Transplantation. 2013;96(3):274–281. doi:10.1097/tp.0b013e31829807d1 23778649

[23] Smail N Tchervenkov J Paraskevas S, . Impact of early graft function on 10-year graft survival in recipients of kidneys from standard- or expanded-criteria donors. Transplantation. 2013;96(2):176–181. doi:10.1097/tp.0b013e318297443b 23765113

[24] Mallon DH Summers DM Bradley JA Pettigrew GJ. Defining delayed graft function after renal transplantation: simplest is best. Transplantation. 2013;96(10):885–889. doi:10.1097/tp.0b013e3182a19348 24056620

[25] Orandi BJ James NT Hall EC, . Center-level variation in the development of delayed graft function after deceased donor kidney transplantation. Transplantation. 2015;99(5):997–1002. doi:10.1097/tp.0000000000000450 25340600 PMC4405384

[26] Mise Y Vauthey J-N Zimmitti G, . Ninety-day postoperative mortality is a legitimate measure of hepatopancreatobiliary surgical quality. Ann Surg. 2015;262(6):1071–1078. doi:10.1097/sla.0000000000001048 25590497 PMC4633391

[27] Joung RH-S Merkow RP. Is it time to abandon 30-day mortality as a quality measure? Ann Surg Oncol. 2021;28(3):1263–1264. doi:10.1245/s10434-020-09262-3 33393040 PMC8148608

[28] Stevens PE Levin A. Evaluation and management of chronic kidney disease: synopsis of the kidney disease: improving global outcomes 2012 clinical practice guideline. Ann Intern Med. 2013;158(11):825–830. doi:10.7326/0003-4819-158-11-201306040-00007 23732715

[29] Fritsche L Hoerstrup J Budde K, . Accurate prediction of kidney allograft outcome based on creatinine course in the first 6 months posttransplant. Transplant Proc. 2005;37(2):731–733. doi:10.1016/j.transproceed.2004.12.067 15848516

[30] Hassanain M Tchervenkov JI Cantarovich M, . Recovery of graft function early posttransplant determines long-term graft survival in deceased donor renal transplants. Transplant Proc. 2009;41(1):124–126. doi:10.1016/j.transproceed.2008.10.046 19249494

[31] Moore J He X Shabir S, . Development and evaluation of a composite risk score to predict kidney transplant failure. Am J Kidney Dis. 2011;57(5):744–751. doi:10.1053/j.ajkd.2010.12.017 21349620

[32] Schnitzler MA Lentine KL Axelrod D, . Use of 12-month renal function and baseline clinical factors to predict long-term graft survival: application to BENEFIT and BENEFIT-EXT trials. Transplantation. 2012;93(2):172–181. doi:10.1097/tp.0b013e31823ec02a 22198496

[33] Wan SS Cantarovich M Mucsi I Baran D Paraskevas S Tchervenkov J. Early renal function recovery and long-term graft survival in kidney transplantation. Transplant Int. 2016;29(5):619–626. doi:10.1111/tri.12775 26988072

[34] Levey AS Stevens LA Schmid CH, . A new equation to estimate glomerular filtration rate. Ann Intern Med. 2009;150(9):604–612. doi:10.7326/0003-4819-150-9-200905050-00006 19414839 PMC2763564

[35] Bouwmeester W Moons KG Kappen TH, . Internal validation of risk models in clustered data: a comparison of bootstrap schemes. Am J Epidemiol. 2013;177(11):1209–1217. doi:10.1093/aje/kws396 23660796

[36] Butala NM Reese PP Doshi MD Parikh CR. Is delayed graft function causally associated with long-term outcomes after kidney transplantation? Instrumental variable analysis. Transplantation. 2013;95(8):1008–1014. doi:10.1097/tp.0b013e3182855544 23591726 PMC3629374

[37] Yarlagadda SG Coca SG Formica RN Jr. Poggio ED Parikh CR. Association between delayed graft function and allograft and patient survival: a systematic review and meta-analysis. Nephrol Dial Transplant. 2008;24(3):1039–1047. doi:10.1093/ndt/gfn667 19103734

[38] Phillips BL Ibrahim M Greenhall GHB Mumford L Dorling A Callaghan CJ. Effect of delayed graft function on longer-term outcomes after kidney transplantation from donation after circulatory death donors in the United Kingdom: a national cohort study. Am J Transplant. 2021;21(10):3346–3355. doi:10.1111/ajt.16574 33756062

[39] Lee J Song SH Lee JY, . The recovery status from delayed graft function can predict long-term outcome after deceased donor kidney transplantation. Sci Rep. 2017;7(1):13725. doi:10.1038/s41598-017-14154-w 29057921 PMC5651849

[40] Tapiawala SN Tinckam KJ Cardella CJ, . Delayed graft function and the risk for death with a functioning graft. J Am Soc Nephrol. 2010;21(1):153–161. doi:10.1681/ASN.2009040412 19875806 PMC2799285

[41] Infante B Franzin R Madio D, . Molecular mechanisms of AKI in the elderly: from animal models to therapeutic intervention. J Clin Med. 2020;9(8):2574. doi:10.3390/jcm9082574 32784471 PMC7464895

[42] Cavaille-Coll M Bala S Velidedeoglu E, . Summary of FDA workshop on ischemia reperfusion injury in kidney transplantation. Am J Transplant. 2013;13(5):1134–1148. doi:10.1111/ajt.12210 23566221

[43] Poesen R Bammens B Claes K, . Prevalence and determinants of anemia in the immediate postkidney transplant period. Transplant Int. 2011;24(12):1208–1215. doi:10.1111/j.1432-2277.2011.01340.x 21929730

[44] Sandal S Luo X Massie AB Paraskevas S Cantarovich M Segev DL. Machine perfusion and long-term kidney transplant recipient outcomes across allograft risk strata. Nephrol Dial Transplant. 2018;33(7):1251–1259. doi:10.1093/ndt/gfy010 29474675 PMC6030984

[45] Van Houwelingen HC. Dynamic prediction by landmarking in event history analysis. Scand J Stat. 2007;34(1):70–85. doi:10.1111/j.1467-9469.2006.00529.x

