# Supplementary material for: Predicting Long-term Outcomes in Deceased Donor Kidney Transplant Recipients Using Three Short-term Graft Characteristics
Source: Kidney360. 2023 May 22;4(6):e809–16. doi: 10.34067/KID.0000000000000154 (PMC10371380; doi:10.34067/KID.0000000000000154)
Supplement: SUPPLEMENTARY MATERIAL [file kidney360-4-e809-s001.pdf]

Supplementary table 1: Baseline characteristics stratified by 90-day renal function recovery

	>100 (n=471)	75-100 (n=179)	≤75 (n=142)
**Recipient characteristics**			
Age, mean (SD)	54.6 (13.6)	56.1 (12.5)	57.3 (11.9)
Female sex, %	31.2	40.8	35.2
BMI in kg/m^2, mean (SD)	26.4 (5.3)	27.3 (4.5)	28.6 (5)
White race, %	65.2	73.2	73.2
Type of dialysis, %			
Hemodialysis	83.6	78.8	76.8
Peritoneal dialysis	7.9	5.0	5.6
Pre-emptive	7.0	15.6	16.9
Missing	1.5	0.6	0.7
Months on dialysis, mean (SD)	18.4 (9.9)	10.6 (6.9)	19.0 (17.4)
Cause of end-stage renal disease, %			
Diabetes	24.8	27.3	24.6
Polycystic kidney disease	18.4	13.1	11.7
Glomerulonephritis	30.5	26.7	33.3
Hypertension	8.5	12.5	11.2
Other	17.7	20.5	11.2
Cold ischemia time, hrs (SD)	16.4 (6.4)	17 (7.1)	17.9 (6.5)
Warm ischemia time, hrs (SD)	0.8 (0.3)	0.8 (0.3)	0.8 (0.2)
Machine perfusion, %	44.2	51.4	56.3
**Donor characteristics**			
Age, mean (SD)	51.1 (14.8)	53.2 (14.6)	57.6 (12.9)
Female sex, %	40.3	55.3	58.5
BMI, mean (SD)	27.1 (5.1)	25.7 (4.7)	29.0 (17.8)
White race, %	84.3	83.8	77.5
Expanded criteria donor, %	37.4	41.9	57.8
Donation after cardiac death, %	5.9	7.8	12.7
Terminal eGFR, mean (SD)	104.3 (17.7)	106.4 (18.2)	94.8 (22.3)
Induction Immunosuppression, %			
Anti-thymocyte globulin	57.5	54.2	51.4
Alemtuzumab	30.4	38.6	39.4
Interleukin-2 receptor inhibitor	8.9	7.3	10.6
Maintenance Immunosuppression, %			
Tacrolimus	85.1	85.5	78.9
Cyclosporine	7.4	8.9	9.9
Sirolimus	0.9	1.7	2.1
Mycophenolate	84.7	85.5	83.8
Azathioprine	5.1	5.6	4.2
Prednisone	64.8	55.3	52.1
Renal function recovery, mean (SD)	143.3 (43.3)	89.0 (7.4)	61.3 (12.5)
90-day eGFR <30, %	0.0	1.7	36.6
DGF, %	21.4	21.8	38.0

BMI: body mass index, DGF: delayed graft function, eGFR: estimated glomerular filtration rate, SD: standard deviation

Supplementary table 2: Baseline characteristics stratified by 90-day estimated glomerular filtration rate

	eGFR ≥30 (n=737)	eGFR<30 (n=55)
**Recipient characteristics**		
Age, mean (SD)	55.2 (13.1)	59.4 (11.7)
Female sex, %	85.5	67.3
BMI in kg/m^2, mean (SD)	26.9 (5.1)	28.9 (5.3)
White race, %	67.3	85.5
Type of dialysis, %		
Hemodialysis	80.5	92.7
Peritoneal dialysis	7.3	0
Pre-emptive	11.0	7.3
Missing	1.2	0
Months on dialysis, mean (SD)	17.4 (14.7)	16.7 (6.0)
Cause of end-stage renal disease, %		
Diabetes	27.3	25.1
Polycystic kidney disease	16.4	13.0
Glomerulonephritis	25.0	31.7
Hypertension	9.1	11.2
Other	21.8	19.0
Cold ischemia time, hrs (SD)	16.7 (6.6)	18.9 (6.4)
Warm ischemia time, hrs (SD)	0.78 (0.32)	0.84 (0.22)
Machine perfusion, %	63.0	48.3
**Donor characteristics**		
Age, mean (SD)	52.1 (14.5)	61.3 (11.5)
Female sex, %	61.8	45.9
BMI, mean (SD)	26.9 (5.1)	28.9 (5.3)
White race, %	95.7	96.4
Expanded criteria donor, %	40.5	69.1
Donation after cardiac death, %	7.3	7.6
Terminal eGFR, mean (SD)	99.7 (21.5)	92.4 (18.1)
Induction Immunosuppression, %		
Anti-thymocyte globulin	45.5	56.5
Alemtuzumab	47.3	32.8
Interleukin-2 receptor inhibitor	7.3	9.0
Maintenance Immunosuppression, %		
Tacrolimus	78.2	84.5
Cyclosporine	9.1	8.1
Sirolimus	1.8	1.2
Mycophenolate	78.2	85.2
Azathioprine	7.3	4.9
Prednisone	43.6	61.6
90-day renal function recovery, mean (SD)	121.2 (45.9)	50.9 (14.4)
DGF, %	22.0	58.2

BMI: body mass index, DGF: delayed graft function, eGFR: estimated glomerular filtration rate, SD: standard deviation

Supplementary table 3: Logistic odds ratio for CKD stage ≥4 by various exposures of interest

Variable	1-year		5-year		10-year	
	Univariate	Multivariate[1]	Univariate	Multivariate[1]	Univariate	Multivariate[1]
DGF						
No DGF	1.00	1.00	1.00	1.00	1.00	1.00
DGF	**2.51 (1.73-3.64)**	**2.14 (1.43-3.19)**	**3.38 (2.16-5.32)**	**2.95 (1.84-4.71)**	**3.75 (2.12-6.64)**	**2.94 (1.56-5.53)**
90-day RFR						
>100	1.00	1.00	1.00	1.00	1.00	1.00
75-100	**1.71 (1.11-2.63)**	**2.00 (1.26-3.18)**	**1.79 (1.12-2.88)**	2.04 (1.23-3.37)	**1.93 (1.08-3.43)**	1.68 (0.85-3.32)
<75	**4.30 (2.79-6.61)**	**5.94 (3.64-9.66)**	**4.45 (2.57-7.70)**	**5.76 (3.17-10.5)**	**4.91 (2.38-10.15)**	**3.93 (1.75-8.84)**
90-day eGFR						
≥30	1.00	1.00	1.00	1.00	1.00	1.00
<30	**19.74 (9.03-43.2)**	**22.1 (9.50-52.46)**	**59.5 (8.02-441.1)**	**49.9 (65.2-381.9)**	--	
Risk Score						
None	1.00	1.00	1.00	1.00	1.00	1.00
Low	1.56 (0.93-2.61)	1.13 (0.64-1.97)	**2.27 (1.28-4.00)**	**1.78 (0.98-3.28)**	**2.25 (1.14-4.42)**	1.88 (0.90-3.97)
Medium	**2.95 (1.76-4.96)**	**2.41 (1.37-4.26)**	**6.52 (3.2-13.28)**	**5.17 (2.45-10.92)**	**8.67 (2.92-25.70)**	**6.93 (2.17-22.16)**
High	**121.2 (16.3-903.0)**	**146.7(18.8-975.3)**	**46.95 (6.18-356.8)**	**40.13 (5.07-317.8)**	-	-

1 Adjusted for recipient age, sex, race, BMI, type of dialysis, history of hypertension and diabetes, and machine perfusion, donor age and donor sex

DGF: delayed graft function, eGFR: estimated glomerular filtration rate, RFR: renal function recovery

Supplementary table 4: Baseline characteristics of the external validation cohort

	Total cohort (n=672)
**Recipient characteristics**	
Age, mean (SD)	50.9 (13.1)
Female sex, %	38.2
BMI in kg/m^2, mean (SD)	26.0 (4.7)
White race, %	80.4
Type of dialysis, %	
Hemodialysis	NA
Peritoneal dialysis	NA
Pre-emptive	13.8
Missing	
Months on dialysis, mean (SD)	44.4
Cause of end-stage renal disease, %	
Diabetes	14.1
Polycystic kidney disease	17.1
Glomerulonephritis	40.0
Hypertension	8.2
Other.missing	20.6
Machine perfusion, %	29.0
**Donor characteristics**	
Age, mean (SD)	50.3 (13.7)
Female sex, %	49.7
BMI, mean (SD)	27.0 (5.4)
Expanded criteria donor, %	33.4
Donation after cardiac death, %	7.6
